# Draft Genome Sequence of an Isolate of Genotype VII Newcastle Disease Virus Isolated from an Outbreak in Fighting Cock in Peru

**DOI:** 10.1128/mra.01293-22

**Published:** 2023-01-31

**Authors:** Manolo Fernández-Díaz, Angela Montalván-Avalos, Gisela Isasi-Rivas, Doris Villanueva-Pérez, Stefany Quiñones-Garcia, Luis Tataje-Lavanda, Dora Rios-Matos, Milagros Lulo-Vargas, Manolo Fernández-Sánchez, Luis A. Guevara-Sarmiento, Mirko Zimic, Aldo Rojas-Neyra, Katherine Calderón

**Affiliations:** a Laboratorios de Investigación y Desarrollo, Farmacológicos Veterinarios S.A.C., Chincha Alta, Ica, Peru; Katholieke Universiteit Leuven

## Abstract

This study presents a draft genome sequence of a Newcastle disease virus (NDV) strain (VFAR-136) isolated from a fighting cock (Gallus gallus) in the south of Peru. Strain VFAR-136 is a new report of NDV genotype VII circulating in Peru.

## ANNOUNCEMENT

Newcastle disease (ND) is an avian viral disease that affects the poultry industry worldwide. ND is caused by Newcastle disease virus (NDV), recently known as *Avian orthoavulavirus 1* (AOAV-1); it belongs to the genus *Orthoavulavirus* in the family *Paramyxoviridae* and has a negative-sense single-stranded RNA (−ssRNA) genome (1). According to molecular analysis, NDV strains have been classified as class I (single genotype) or class II (genotypes I to XXI). The class I strains are avirulent, while class II strains are generally lentogenic, mesogenic, or velogenic (2). In recent years, only subgenotype XIIa has been reported as circulating in Peru (3–5).

In this study, an NDV outbreak in fighting cocks (Gallus gallus) was detected in 2022 in Chincha, Peru. Viral isolation was performed in 9-day-old specific-pathogen-free (SPF) embryonated chicken eggs from a tracheal swab (6). Viral RNA (500 ng) from the isolated virus (VFAR-136) was extracted using the QIAamp viral RNA minikit (Qiagen), double-stranded cDNA was synthesized, and a library was prepared using the SQK-DCS109 kit (Oxford Nanopore Technologies). The double-stranded cDNA was prepared with SuperScript IV reverse transcriptase (Invitrogen), LongAmp *Taq* 2× master mix (New England), and PR2 primer (Oxford Nanopore Technologies) (7). End prep repair and dA-tailing were performed using Ultra II end prep enzyme mix (New England Biolabs). The sample was ligated to the flow cell adapter with blunt/TA ligase master mix (New England Biolabs). Purification steps were performed using AMPure XP beads (Beckman Coulter). A portion (50 ng) of the library (prepared using the SQK-DCS109 kit) was loaded into a FLO-MIN106 (R9.4) flow cell. The sample was sequenced on a MinION Mk1b device, using the Fast base-calling model, with MinKNOW software v.22.08.8. After base calling with Guppy v.6.37 using the high-accuracy model, the fastq files were analyzed using the Galaxy Web platform (https://usegalaxy.eu/) (8). Adapters were removed using Porechop (Galaxy v.0.2.4+galaxy0). The remaining reads were assembled *de novo* using rnaviralSPAdes (Galaxy v.3.15.4+galaxy2), Flye (Galaxy v.2.9+galaxy0), MEGAHIT (Galaxy v.1.2.9+galaxy0), and finally, WTDBG (Galaxy v.1.2.8.1). We trimmed the 5′ end because it had a low average read depth (<20×). Alignment and phylogenetic analysis were conducted using MAFFT on XSEDE v.7.490 (9) and the MEGA11 v.11.013 program (10).

**FIG 1:**
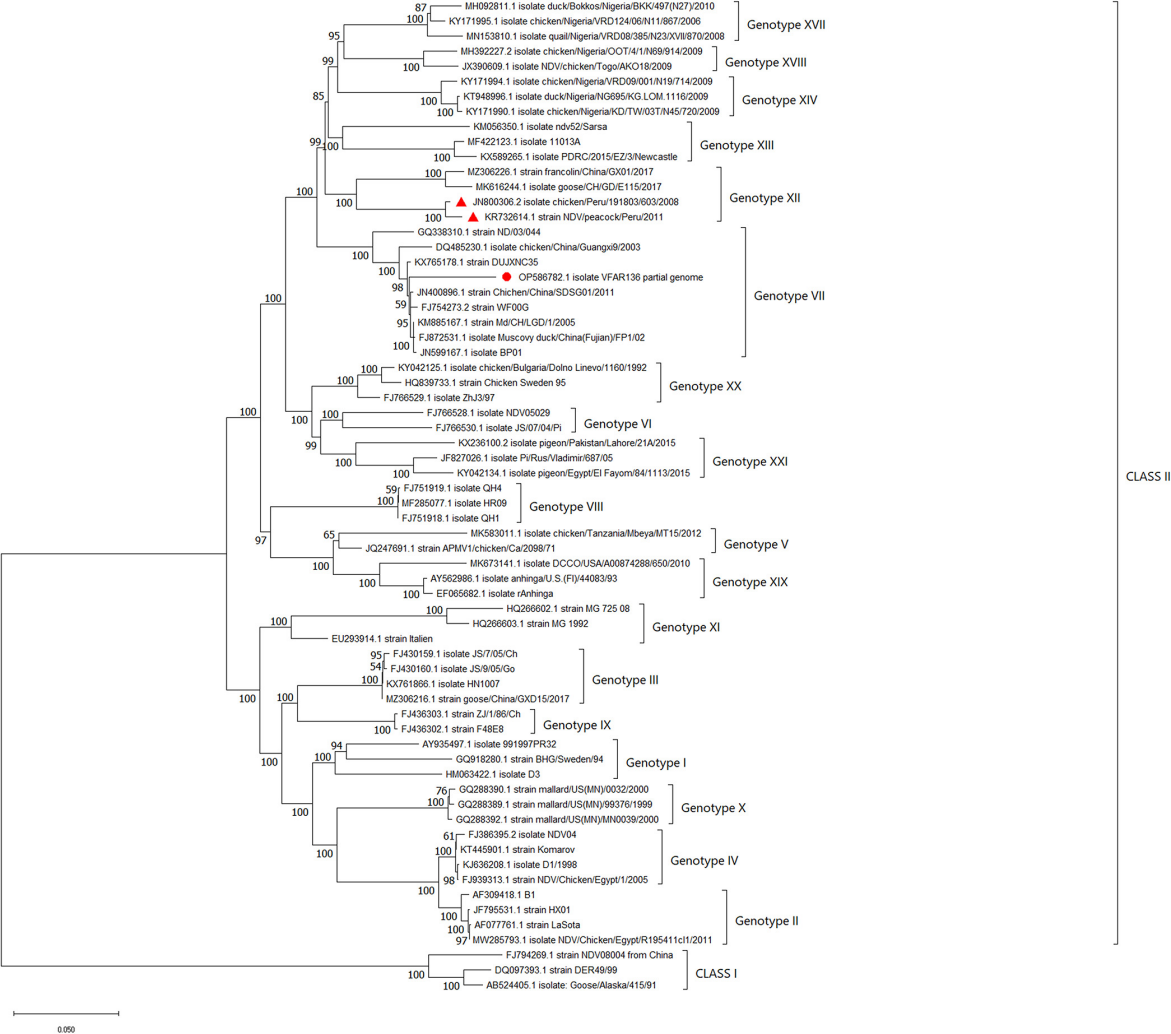
Phylogenetic tree constructed using the neighbor-joining method with 1,000 bootstraps. Evolutionary distances were calculated using the Tamura-Nei method. The rate variation among sites was modeled with a gamma distribution of 1. Gaps and missing data were discarded (complete deletion option). A total of 10,942 positions were analyzed. The analysis was performed using the MEGA11 program. The new NDV genotype VII isolate from the current study (VFAR-136) is indicated with a red circle. The NDV strains isolated from Peru in the other studies are marked with red triangles.

Genome sequencing generated 356,400 reads, comprising 6.41 Gb with an *N*_50_ value of 1.65 kb. Assembly generated a viral genome of 11,041 nucleotides (nt) (average depth, 45.46×), with 45% G+C content. Local alignment using BLASTn showed high similarity (95%) with other NDV genotype VII strains (±95%), such as Chicken/China/SDSG01/2011 (GenBank accession no. JN400896.1), Md/CH/LGD/1/2005 (KM885167.1), Muscovy duck/China (Fujian)/FP1/02 (FJ872531.1), and BP01 (JN599167.1). Phylogenetics analysis based on the whole genome placed strain VFAR-136 within the genotype VII clade (100% bootstrap) (Fig. 1). The analysis suggested that strain VFAR-136 represents a virulent pathotype due to the presence of a polybasic amino acid motif (^112^RRQKR↓F^117^) in the F protein cleavage site, which is referred to as a key determinant of virulence (11). The genomic sequence of strain VFAR-136 will be important for the development of homolog vaccines against genotype VII strains circulating in Peru and for epidemiological surveillance studies.

### Data availability.

The whole-genome sequence of strain VFAR-136 has been deposited at GenBank under accession no. OP586782. The version described in this paper is the first version. The raw data were submitted to the SRA under accession no. PRJNA914773.
